# Enhancing health and wellness by, for and with Indigenous youth in Canada: a scoping review

**DOI:** 10.1186/s12889-022-14047-2

**Published:** 2022-08-29

**Authors:** Udoka Okpalauwaekwe, Clifford Ballantyne, Scott Tunison, Vivian R. Ramsden

**Affiliations:** 1 Health Sciences Program, College of Medicine, University of Saskatchewan, Saskatoon, Saskatchewan S7N 5E5 Canada; 2 Sturgeon Lake Youth Center, Sturgeon Lake First Nation, Sturgeon Lake, Saskatchewan S0J 2E1 Canada; 3 University of Saskatchewan, Saskatoon, Saskatchewan S7N 0X1 Canada; 4 Research Division, Department of Academic Family Medicine, University of Saskatchewan, Saskatoon, Saskatchewan S7M 3Y5 Canada

**Keywords:** Indigenous youth, Health, Wellness, Authentic engagement, Culture as treatment, Wellness promotion

## Abstract

**Background:**

Indigenous youth in Canada face profound health inequities which are shaped by the rippling effects of intergenerational trauma, caused by the historical and contemporary colonial policies that reinforce negative stereotypes regarding them. Moreover, wellness promotion strategies for these youth are replete with individualistic Western concepts that excludes avenues for them to access holistic practices grounded in their culture. Our scoping review explored strategies, approaches, and ways health and wellness can be enhanced by, for, and with Indigenous youth in Canada by identifying barriers/roadblocks and facilitators/strengths to enhancing wellness among Indigenous youth in Canada.

**Methods:**

We applied a systematic approach to searching and critically reviewing peer-reviewed literature using the Preferred Reporting Items for Systematic Reviews and Meta-Analyses extension for Scoping Reviews [PRISMA-ScR] as a reporting guideline. Our search strategy focused on specific keywords and MeSH terms for three major areas: Indigenous youth, health, and Canada. We used these keywords, to systematically search the following electronic databases published in English between January 01, 2017, to May 22, 2021: Medline [Ovid], PubMed, ERIC, Web of Science, Scopus, and iportal. We also used hand-searching and snowballing methods to identify relevant articles. Data collected were analysed for contents and themes.

**Results:**

From an initial 1695 articles collated, 20 articles met inclusion criteria for this review. Key facilitators/strengths to enhancing health and wellness by, for, and with Indigenous youth that emerged from our review included: promoting culturally appropriate interventions to engage Indigenous youth; using strength-based approaches; reliance on the wisdom of community Elders; taking responsibility; and providing access to wellness supports. Key barriers/roadblocks included: lack of community support for wellness promotion activities among Indigenous youth; structural/organizational issues within Indigenous communities; discrimination and social exclusion; cultural illiteracy among youth; cultural discordance with mainstream health systems and services; and addictions and risky behaviours.

**Conclusion:**

This scoping review extracted 20 relevant articles about ways to engage Indigenous youth in health and wellness enhancement. Our findings demonstrate the importance of promoting health by, and with Indigenous youth, by engaging them in activities reflexive of their cultural norms, rather than imposing control measures that are incompatible with their value systems.

**Supplementary Information:**

The online version contains supplementary material available at 10.1186/s12889-022-14047-2.

## Introduction

The term ‘Indigenous’ is internationally recognized to describe a distinct group of people that live within or are attached to geographically distinct ancestral territories [1, 2]. In Canada, the term Indigenous is an inclusive term used to refer to the First Nations, Métis, and Inuit people, each of which has unique histories, cultural traditions, languages, and beliefs [3–5]. Indigenous peoples are the fastest-growing population in Canada, with a population estimated at 1.8 million, which is 5.1% of the Canadian population [6, 7]. Within this population, 63% identify as First Nation, 33% as Métis, and 4% as Inuit [6, 7]. Indigenous youth are the youngest population in Canada, with over 50% of Indigenous youth under 25 years [7]. Projections of Indigenous peoples in Canada have estimated a 33.3 to 78.7% increase in Indigenous populations, with the youth making up the largest proportion of the Indigenous population by 2041 [6, 7].

Before European contact in North America, Indigenous peoples in Canada lived and thrived with their cultures, languages, and distinct ways of knowing [2]. However, Indigenous peoples in Canada rank lower in almost every health determinant when compared with non-Indigenous Canadians [8–10]. A report on health disparities in Saskatoon, Saskatchewan, described First Nations peoples to be “more likely to experience poor health outcomes in essentially every indicator possible” (page 27) [11]. This greater burden of ill health among Indigenous peoples in Canada has been attributed to systemic racism (associated with differences in power, resources, capacities, and opportunities) [9, 10, 12, 13] and intergenerational trauma (stemming from the past and ongoing legacy of colonization such as experienced through the Indian residential and Day school systems, the Sixties Scoop, and the ongoing waves of Indigenous child and youth apprehensions seen in the foster and child care structures that remove Indigenous children from their family, community and traditional lands) [3, 9, 10, 12–17]. These traumatic historical events, along with ongoing inequities, such as: socioeconomic and environmental dispossession; loss of language; disruption of ties to Indigenous families, community, land and cultural traditions; have been reported to exacerbate drastically and cumulatively the physical, mental, social and spiritual health of Indigenous peoples in Canada, creating “soul wounds” (3 p.208) that require interventions beyond the Westernized biomedical models of health and healing [3, 9, 10, 12–21].

In the same way, Indigenous youth in Canada face some of the most profound health inequities when compared with non-Indigenous youth which can be further shaped by the rippling effects of intergenerational trauma caused by the historical and contemporary colonial policies that reinforce or legitimize negative stereotypes regarding Indigenous youth in Canada [2, 10, 14, 20, 22–27]. When compared with their non-Indigenous peers, Indigenous youth in Canada have been reported to be more likely to have higher rates of chronic conditions [e.g., diabetes, obesity, chronic respiratory diseases, heart diseases, etc.] [14], discrimination [28, 29], youth incarceration and state care [12, 20, 30], poverty [31], homelessness [32], higher adverse mental health conditions [20, 33–37], higher suicide rates [33, 38, 39], and lower overall life expectancies [24, 40–42].

Indigenous peoples’ perception of health and wellness is shaped by their worldview and traditional knowledge [43, 44]. While the Western concept of health broadly defines health as the state of complete physical, mental, social well-being, and not merely the absence of disease [45], Indigenous peoples understand health in a holistic way [26] that seeks balance between the physical, mental, emotional, and spiritual aspects of an Indigenous person in reciprocal relationships with their families, communities, the land, the environment, their ancestors, and future generations [46–48]. Unfortunately, this holistic concept of health and wellness opposes the individualistic and biomedically focused Western worldview of health, which is a dominant lens commonly used in health research, projects, and programs involving Indigenous communities [46]. This practice further perpetuates the legacy of colonization and excludes avenues for Indigenous communities to access holistic healing practices “grounded in their culture” [43, 49, 50]. For example, health research involving Indigenous peoples in Canada tends to focus on Indigenous health deficits and identified social determinants in the communities, more often and without proper representation [43]. Additionally, there is the imposition of research *on* rather than *with* youth [43, 44]; and the failure to acknowledge Indigenous worldviews in research, to ensure in benefits them [43].

Authentically engaging with Indigenous youth has been cited by Indigenous scholars as one of the ways of achieving and enhancing wellness by, for, and with youth [51, 52]. This is characterized by meaningful and sustained involvement of the youth in program planning, development, and decision-making to promote self-confidence and positive relationships [53]. Authentic engagement involves working *with* rather than *on* youth as research partners or program planning participants [54]. This shift to working *with* rather than *on* implies respect for the knowledge of the lived experiences of the youth involved [54–56] and is based on meaningful relationships built over time among all involved [53, 57, 58]. Research has shown that engaging youth (Indigenous or non-Indigenous) as partners in a project/program fosters a sense of belonging, self-determination, and self-actualization within their community; thus, enhancing community wellness [54, 56, 58, 59].

This paper explores what is known in the peer-reviewed literature about strategies, approaches, and ways to engage Indigenous youth in health and wellness enhancement. Our main objective is to use information gathered from this review to inform youth engagement strategies, by considering the facilitators/strengths and barriers/roadblocks to enhancing wellness with Indigenous youth. We define facilitators in this context as factors that improve, enhance, strengthen, or motivate a journey to health, wellness, and self-determination. These are considered ‘strengths’ in the language of Indigenous peoples as they support equitable strength-based pathways towards reconciliation. Conversely, barriers are roadblocks, and demotivating factors or processes that limit and challenge Indigenous peoples’ access to achieving health and wellness. Our overarching research question was, *in what ways can Indigenous youth enhance health and wellness for themselves, their family, and the Indigenous communities where they live?*

Sub-questions included:

- *What factors do Indigenous youth in Canada identify as facilitators/strengths to enhancing health and wellness?*
- *What factors do Indigenous youth in Canada identify as barriers/roadblocks to enhancing health and wellness?*

## Methodology and methods

Scoping reviews help provide an overview of the research available on a given area of interest where evidence is emerging [60]. While there are several accepted approaches to such reviews, this scoping review was undertaken using the Joanna Briggs Institute (JBI) Guideline for scoping reviews [61]. This approach was based on the Arksey and O’Malley methodological framework [62], which was further advanced by Levac et al. [60], and Peter et al. [61]. Our search strategy focused on primary sources that elucidated youth-driven, youth-led, or youth-engaged strategies carried out by, for, and with Indigenous youth to enhance health and wellness. We chose to explore all health programs and research inquiry that explore health challenges on the physical, mental, emotional, and spiritual aspects of an Indigenous person to encompass the definition of health and wellness as defined and understood from an Indigenous perspective. This scoping review is reported in accordance with the guidelines provided in the Preferred Reporting Items for Systematic Reviews and Meta-Analyses (PRISMA) extension for Scoping Reviews (PRISMA-ScR) [63]. See Supplementary material file 1 for PRISMA-SCR checklist.

### Protocol registration and reporting information

There was no pre-published or registered protocol before the commencement of this study.

### Eligibility criteria

#### Types of studies

A priori inclusion criteria for articles in this study included: 1] peer-reviewed journal articles reporting health and wellness programs, initiatives, and/or strategies among Indigenous youth in Canada, and 2] peer-reviewed journal articles published between January 01, 2017, to May 22, 2021. We chose a 5-year time frame to limit our findings to the most updated peer-reviewed literature which could provide implications for the growing body of work done in the field of Indigenous research among youth. Systematic reviews, meta-analyses, study protocols, opinion pieces, and narrative reviews were excluded.

#### Participants

Peer-reviewed studies involving Indigenous youth (First Nations, Métis, and Inuit) in *Canada* were eligible for inclusion. We considered the fluidity of definitions for youth by age range as literature sources generally defined youth in stages between adolescence to early adulthood [6, 64, 65]. In Canada, the Government of Canada uses several age brackets to identify youth depending on context, program, or policies in question. For example, Statistics Canada defines youth as between 15 to 29 years [6], Health Canada in the first State of Youth Report defined youth as between 12 to 30 years [65] when referring to statistical reports, and as between 13 to 36 years when referring to youth-led programs and policies [65]. However, for the purposes of this review we defined and referred to Indigenous youth or young people as between 10 to 24 years to be more representative of a broader definition of youth which is in keeping with Indigenous peoples’ worldviews, languages, and cultures and more representative of a broader definition of youth as offered by Sawyer et al. [64].

### Information sources and search strategy

With the assistance of an Academic Reference Librarian, search terms were identified, which were categorized and combined into three conceptual MeSH terms that we adapted for the database-specific search strategy. These terms included: Indigenous youth (including synonyms and MeSH terms), health (including synonyms and MeSH terms) and Canada. Thus, studies were then identified for this scoping review by searching electronic databases and hand-searching reference lists of included articles.

Initially, the following databases (Medline (Ovid), PubMed, ERIC, Web of Science and Scopus) were used to identify relevant articles published between January 1, 2017, and April 30, 2021. This constituted our first search. We then carried out a second search (updated search) on May 22, 2021, using the same search queries on the same library databases; in addition, we included the University of Saskatchewan’s Indigenous Studies Portal (iPortal) [66] to ensure we had as many hits as possible for our search query on focused studies with Indigenous communities. To ensure exhaustiveness, we employed hand-searching techniques and snowballing methods to identify articles relevant to the research questions by reviewing reference lists of relevant articles that met the eligibility criteria. Following this, all the identified articles were collated in Endnote Reference Manager version X9.3 [67] and exported, after removing duplicates, into Distiller SR [68], a web-based systematic review and meta-analysis software. The syntax used on electronic databases and the University of Saskatchewan’s iPortal to identify potentially relevant articles for inclusion into this review study is outlined in Table 1.

**Table 1:** Keyword search syntax used for library search

1. **Indigenous youth/** 2. Indigenous adj3 youth OR Indigenous adj3 adolescent OR Cree adj2 youth OR Cree adj2 adolescent OR Indigenous adj3 communit$ OR Indigenous adj2 reserv$ OR reserv$ OR Aborigine OR Aboriginal OR Indigenous OR Native$ OR Indigen$ OR First adj1 Nation$ OR Métis$ OR Inuit$ OR Inuk$.ti.ab 3. **Health/** 4. Health OR wellness OR health adj2 promotion OR mental adj2 health OR mental adj2 health adj3 wellness OR physical adj2 health OR spiritual adj2 health OR emotional adj2 health OR holistic adj2 health OR medicine adj2 wheel.ti.ab 5. **Canada/** 6. Canada OR Alberta OR British adj1 Columbia OR Manitoba OR New adj1 Brunswick OR Newfoundland adj1 and abj1 Labrador OR Northwest adj1 Territor$ OR Nova adj1 Scotia OR Nunavut OR Ontario OR Prince adj1 Edward adj1 Island OR Quebec OR Saskatchewan OR Yukon.ti.ab 7. #2 AND #4 AND #6	

### Selection of sources of evidence

Two iterative stages were employed to select sources of evidence for this review study. First, we created screening, coding, and data extraction forms using Distiller SR [68] for each stage. In the first stage, UO screened titles and abstracts of all articles using the following keywords: Indigenous youth; health; wellness; engagement and Canada. In the second stage, UO independently screened and reviewed the full-text articles (FTAs) of citations included from the first stage. The questions in Table 2 were used to screen the eligibility for inclusion of the article for data extraction. A second reviewer (ST) also independently reviewed and screened every 10th FTA citation from the first phase to check inter-rater reliability.

**Table 2:** Full-text articles screening form used on DistillerSR

1. Did the study objective(s) focus on health and wellness promotion? (Yes/No/Unsure) 2. Did the study focus on Indigenous communities? (Yes/No/Unsure) 3. Did the study focus on Indigenous youth? (Yes/No/Unsure) 4. Were youth engaged in *some way* in the study? (Yes/No/Unsure) 5. Did youth lead or co-lead in the study? (Yes/No/Unsure) 6. Were outcomes derived (or discussed) in the study? (Yes/No/Unsure)	

### Data charting process and data items

Data were extracted using a pre-designed data extraction form on DistillerSR [68]. All extracted data were exported into Microsoft Excel [69] for data cleaning and analysis. The title fields used to extract data from included articles are shown in Table 3.

**Table 3:** Data extraction title fields

Author(s)	
Year of Publication	
Province in Canada	
Indigenous Nation focused on (First Nations, Métis, Inuit, or others specified)	
Indigenous community name (if stated)	
Setting: school, Indigenous community, other (list)	
Study objective(s)	
Methods and methodology	
Study type: quantitative, qualitative, mixed-methods study, other (list)	
Study design: case study, cross-sectional, prospective (other than RCT), RCT, retrospective, review study, PAR, narrative, grounded theory, phenomenological study, other (list)	
Youth sample size (if stated)	
Youth age bracket (if stated)	
Data collection methods: structured surveys, semi-structured surveys, focus group discussions, key-informant interviews, storytelling, photovoice, other (list)	
Outcomes	
How was health and wellness enhanced by/with/for youth in the study (describe)	
How were youth engaged in the study (describe)?	
What were barriers to youth wellness enhancement (describe)	
What were facilitators to youth wellness enhancement (describe)	
Methodological limitations and directions for further research (describe)	

### Critical appraisal of individual sources of evidence

Conjointly, UO and CB appraised each article included considering characteristics and methodological quality using the JBI Critical Appraisal Tool for qualitative and quantitative studies [70]. The JBI Critical Appraisal Tool was designed to evaluate the rigour, trustworthiness, relevance, and potential for bias in study designs, conduct, and analysis [70]. Results on the critical appraisals are summarized in Supplementary material file 2.

### Synthesis of results

We categorized findings in this review as facilitators/strengths and barriers/roadblocks to enhancing wellness by, for, and with Indigenous youth, further describing how youth described wellness promotion. We met weekly via videoconference to discuss, review, and revisit our study evaluation protocol to ensure we adhered strictly to the scoping review guidelines.

## Outcomes

### Selection of sources of evidence

**Fig. 1:**
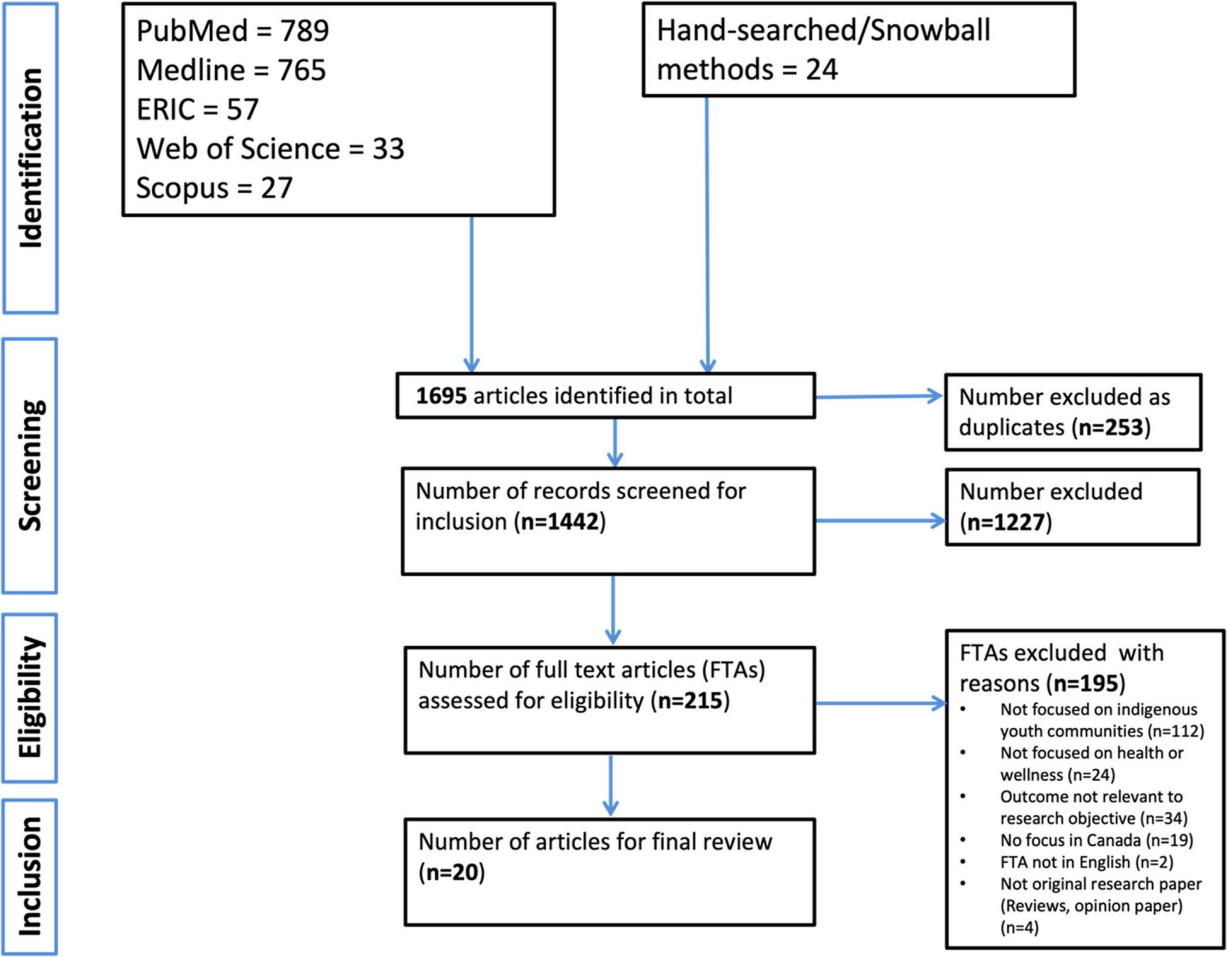
PRISMA flowchart showing selection of articles for scoping review

As a result of our literature search, 1671 articles from five library databases and 24 articles through hand-search and snowball methods were identified. Of the 1695 articles, 253 were excluded as duplicates on EndNote vX9.3 using the ‘remove duplicates’ function on the software. Another 1227 articles were excluded following screening of title and abstracts on Distiller SR which we had fed with a series of screening questions (see Table 2) that were reviewed independently by two reviewers (UO and ST). Inter-rater reliability (Cohen’s kappa) calculated was 0.886, standard error = 0.147, *p*-value = 0.001. Where there were conflicts in article inclusion ratings, a third reviewer (CB), was brought in to discuss and provide a resolution. This left 215 articles for full-text article (FTA) screening. After reviewing 215 FTAs, a further 195 articles were excluded, leaving 20 articles for inclusion into the final review. Articles were excluded in the eligibility stage for the following reasons, 1) articles not focused on Indigenous youth or Indigenous communities, 2) articles not focused on Indigenous health and/or wellness, 3) articles not primarily focused in Canadian settings, 4) articles not written in English, 5) articles considered irrelevant or not applicable to addressing the research objectives or research questions of our study, 6) articles other than original research (i.e., we excluded review studies, opinion papers, and conference abstracts). A flowchart of article selection can be found in Fig. 1.

### Characteristics of sources of evidence

The general and methodological characteristics of all 20 included articles are summarized in Table 4. Of these, one study was published in 2017, two in 2018, eleven in 2019, four in 2020 and two in 2021. Five (25%) studies that were included were set in the province of Ontario, four (20%) in the province of Saskatchewan, three (15%) in the Northwest Territories and two in the province of Alberta. Fifty percent (10/20) of the studies recruited or focused on Indigenous (First Nations, Métis, and Inuit) people as study participants, seven (35%) studies recruited or concentrated on First Nations peoples only, and three (15%), on Inuit peoples only. Sixteen (80%) articles were qualitative studies, three (15%) used mixed methods, and one (5%) was a quantitative study. Eleven (55%) studies used participatory research approaches (which included photovoice, community-based participatory research (CBPR) or participatory action research (PAR)) in their study designs, seven (35%) integrated Indigenous research methods (e.g., the two-eyed seeing approach) into their study design, and five (25%) studies used descriptive or inferential evaluation strategies in their study design. Interviews, focus-group discussions, and discussion circles were the most common data collection methodology used in 17 (85%) of the studies included. Youth were commonly engaged in non-cultural activities in twelve (60%) of the studies and employed a youth-adult co-led strategy in 16 (80%) of the included studies.

**Table 4:** General and methodological characteristics of included studies (*n* = 20)

Publication year	n (%)	Article citations
2017	1 (5.0)	[71]
2018	2 (10.0)	[72, 73]
2019	11 (55.0)	[16, 40, 57, 74–81]
2020	4 (20.0)	[8, 82–84]
2021	2 (10.0)	[5, 44]
Canadian Province/Territory	n (%)	Article citations
Alberta	2 (10.0)	[5, 81]
British Colombia	0 (0.0)	–
Manitoba	1 (5.0)	[76]
New Brunswick	0 (0.0)	–
Newfoundland and Labrador	1 (5.0)	[84]
Nova Scotia	1 (5.0)	[78]
Ontario	5 (25.0)	[16, 44, 71, 72, 75]
Prince Edward Island	1 (5.0)	[80]
Quebec	1 (5.0)	[8]
Saskatchewan	4 (20.0)	[57, 77, 82, 83]
Northwest Territories	3 (15.0)	[73, 74, 79]
Nunavut	1 (5.0)	[40]
Yukon	0 (0.0)	–
Indigenous Nation focus	n (%)	Article citations
First Nations (FN)	7 (35.0)	[8, 16, 57, 72, 78–80]
Métis	–	–
Inuit	3 (15.0)	[40, 74, 84]
Multiple nations (mix of FN, Inuit and/or Métis reported in study)	10 (50.0)	[5, 44, 71, 73, 75–77, 81–83]
Study type	n (%)	Article citations
Qualitative	16 (80.0)	[5, 8, 40, 44, 57, 72–77, 80–83]
Quantitative	1 (5.0)	[16]
Mixed methods	3 (15.0)	[71, 78, 79]
Study design	n (%)	Article citations^a^
PAR, CBPR, Photovoice	11 (55.0)	[8, 40, 44, 57, 72, 74–77, 79, 84]
Ethnography	2 (10.0)	[5, 75]
Cross-sectional study	2 (10.0)	[16, 71]
Evaluation design (Descriptive, or inferential including pre-post implementation design)	5 (25.0)	[73, 74, 78, 81, 84]
Case study	2 (10.0)	[44, 83]
Theoretical	2 (10.0)	[82, 83]
Indigenous research methods	7 (35.0)	[77–83]
Data collection methods	n (%)	Article citations^a^
Interviews, focus groups, discussion circles	17 (85.0)	[5, 8, 40, 44, 57, 71–73, 75–77, 79–84]
Photovoice, visual voice, art-based methods	8 (40.0)	[44, 57, 73, 75, 76, 79, 80, 84]
Semi-structured surveys	4 (20.0)	[5, 73, 74, 78]
Structured surveys	4 (20.0)	[16, 71, 78, 79]
Observations, reflections, fieldnotes	8 (40.0)	[5, 57, 73, 75, 79–81, 83]
Youth engagement	n (%)	Article citations
Youth-led	2 (10.0)	[5, 71]
Youth/adult co-led	16 (80.0)	[8, 40, 44, 57, 72, 73, 75–84]
Adult-led	1 (5.0)	[74]
Not specified	1 (5.0)	[16]
Youth engagement strategies utilized	n (%)	Article citations
Cultural activities (e.g., drumming, singing, dancing, hunting, fishing, etc.)	7 (35.0)	[8, 57, 71, 72, 74, 79, 82]
Non-cultural activities (e.g., non-traditional social and physical activities, including research and training workshops)	12 (60.0)	[5, 40, 44, 73, 75–78, 80, 81, 83, 84]
Not specified	1 (5.0)	[16]

Key: *FN* First Nations, *PAR* Participatory action research, *CBPR* Community-based participatory research

^a^Multiple overlaps for cited studies

### Results of individual sources of evidence

All included studies provided answers relevant to one or more of the research questions with the potential for changing practice and strategies for engagement. All the included studies explored, investigated, or evaluated issues addressing health and wellness among Indigenous youth in Canada. The age range of youth involved in included studies ranged between 11 to 24 years. All studies utilized fun and interactive strategies to engage youth in their respective studies with the outcomes aimed at promoting health, developing capacity in youth participants and engaging youth in collaborating on sustainable outcomes for and with their communities [5, 8, 40, 44, 57, 71–84], save for one [16]. The summary of individual sources of evidence is described in Table 5.

**Table 5:** Characteristics of the included studies [*n* = 20]

S/N	Author(s) [citation]	Objective(s)	Setting	Study type, sample characteristics	Design, Methodology, Methods	Youth engagement strategies	Study Outcomes		Limitations or areas for further research
							Barriers/facilitators to enhancing wellness	How was wellness enhanced?	
1	Anang et al., 2019 [40].	To describe the processes and findings of a community-based participatory research with Inuit youth on suicide prevention	Naujaat, Nunavut, Canada	Qualitative study engaging 36 Inuit youth over three years under the age of 24 years.	CBPR research design integrated with the two-seeing eye framework. Data were collected using interviews and focus group discussions	Youth were engaged in every aspect of the research process as co-researchers.	1)Youth indicated that the processes of engagement to develop actions that reflected their intergenerational cultural traditions facilitated wellness by producing self-pride and self-identity, which was identified as associated with high community youth suicide occurrences. 2)Youth recommended using strength-based approaches to enhance health and wellness within the community	Engaging Inuit youth as co-researchers revitalized an awareness of their cultural identity and produced leadership qualities in the youth involved.	The authors identified the inability to adjust activities with youth availabilities and responsibilities as a limitation in this study.
2	Crooks et al., 2017 [71].	To evaluate the effects of an Indigenous youth-led relationship-focused mentoring program on positive well-being (assessed by mental health and cultural identity).	First Nation, Métis, and Inuit (FNMI) students from a school district in South-Western Ontario, Canada.	Mixed-methods research. 105 FNMI youth between 11 to 14 years.	Cross-sectional study. Data were collected using structured surveys and interviews	Youth were paired with senior classmates and peers in the Fourth R program. This program sought to develop and evaluate school-based, culturally relevant relationship-focused programming with FNMI students. In this program, youth provided peer-mentorship and built relationships through cultural teaching sessions offered to elementary school graders (grade 7 and 8) transitioning to high schools.	Facilitators mentioned: 1) Creation of a culturally sensitive avenue for relationship building and peer-mentorship 2) Because the mentoring program offered participants a culturally sensitive and affirming space to learn about healthy relationships, students embraced their individuality and explored their cultural identity.	The youth described that the mentoring program helped them develop intrapersonal and interpersonal skills and enhanced their knowledge of cultural and healthy relationships. Also, evaluation results showed positive mental health gains after 1 to 2 years of mentorship (1 year qualitatively and 2 years quantitatively)	Limitations reported were; 1) Small sample size of students receiving mentorship. 2) 93% were First Nations ancestry; hence results may not generalize to Métis and Inuit students.
3	Etter et al. 2019 [74].	To describe a community-specific and culturally coherent approach to youth mental health services in a small and remote northern Indigenous community in Canada’s Northwest Territories, under the framework of ACCESS Open Minds (ACCESS OM), a pan-Canadian youth mental health research and evaluation network.	Inuit community of Ulukhaktok, NWT, Canada	Qualitative case report. Youth 18 to 23 years	Participatory approach engaging and training local health workers and ACCESS OM youth workers as leaders and drivers of the programs. Data were collected using semi-structured surveys.	Youth and adults’ connections were strengthened within the Ulukhaktok community as they engaged in events and activities like fishing trips, cooking workshops, arts and craft projects and land-based wellness programs.	Barriers mentioned included: 1) Lack of mental health knowledge or local skills within the community to provide support to youth. 2) Mistrust of mainstream mental health services provided to the community by outside-sourced professionals. 3) General stigma towards mental health by community members.	Wellness was enhanced by empowering the community through training local health workers in mental health using Indigenous-focused modules, providing avenues for cultural connectedness and increased ownership of resources. Additionally, youth connections and the willingness to use mental health support services improved with engagement activities in ways that outside-sourced professional services couldn’t provide	Study limitations reported included; 1)Older youth were less likely to engage due to other domestic priorities (e.g., work, leaving the community, or family priorities) 2) Trained local health workers and youth coordinators expressed the challenge of managing a dual identity that may have influenced community members’ expectations.
4	Flicker et al., 2019 [75].	To describe a strength-based approach to thinking about Indigenous youth HIV prevention activism.	Native Youth Sexual Health Network, Ontario, Canada.	Qualitative study engaging 18 youth between 16 to 24 years.	Ethnography and community-based participatory action research approach using a health promotion framework grounded in the ideas of Indigeneity and decolonization. Data were collected using digital stories shared by youth participants and interviews.	Youth were engaged as leaders and co-researchers in every sphere of the project over three years: from project design to thematic analysis and interpretation. Youth were also engaged in games, movie nights where relationships were fostered, and trust built.	Seven themes were deduced from the thematic analyses by youth. These themes describe both facilitators and barriers to promoting HIV prevention among Indigenous youth. They were: (1) family and elders support, (2) traditional sacred notions of sexuality, (3) the importance of education, (4) reclaiming history, (5) focusing on strength, (6) Indigenous cosmology and (7) overcoming addictions.	Youth described how engaging in this project enhanced their connections to the universe. One youth explained how sharing stories on the sacredness of participating in sweat lodges bolstered his relationships with the creator and the Indigenous understandings of interconnections between living things, including his physical, cultural, and spiritual wellbeing.	Study limitations reported included; 1) Small sample size. 2) Findings should be generalized only to similar contexts and settings.
5	Gaspar et al., 2019 [57].	To build a sense of empowerment among First Nation girls by exploring supports and roadblocks to empowerment in the Girl Power Program.	Sturgeon Lake First Nations, Saskatchewan, Canada	Qualitative study engaging 22 girls between ages 10 to 15 years.	Employed an integrated framework of participatory action research and transformative action research using a strength-based approach that fostered transformative learning environments. Data were collected using storytelling and reflections.	The Girl Power Program was designed to assist girls with mitigating risk factors related to trauma by empowering them to achieve their full potential by integrating Cree cultural teachings, ceremonies, and the rites of passage. Girls in this program were authentically engaged in all aspects of the research processes.	The following were identified as facilitators to empowerment and, by extension, wellness. 1) empowerment programs 2) culture and ceremonies 3) pets 4) sports 5) relationships 6) reading, and 7) kindness Barriers or roadblocks included; 1) Hunger 2) being bullied or abused 3) exposure to drugs and alcohol 4) being smart, and 5)lack of support	Working and *walking* together with the girls on this research facilitated mutual learning and explored ways to identify and implement positive change through sharing stories. The girls indicated that by identifying roadblocks to empowerment, they found healing from wounded spirits, which helped foster positive changes towards wellness through *āhkamēyimowin* (perseverance).	The authors reported that directions for further research should be grounded in culture and contemporary understanding of empowerment while acknowledging the wounded identities and spirits of Indigenous peoples; and the need for co-creating spaces to learn about their spiritual and cultural transitions.
6	Gaudet & Chilton, 2018 [72].	To describe the creation of a youth-centered project and how its re-centers Indigenous values and conception of health and wellbeing	Moose Cree First Nation community, James Bay region, Ontario, Canada.	Qualitative study engaging 6 Indigenous youth, Knowledge Keepers, Elders, and families.	Community-based participatory research approach ground in the Cree philosophy of *milo pimatisiwin*, “good and healthy living.”	Youth were engaged as partners in the project, exploring the significance of sharing *pimatisiwin* teachings over the local youth radio station and within land-based initiatives. Data were collected for evaluation using interviews and focus group discussions	1)The authors and elders described *milo pimatisiwin* (good and healthy living) as a living concept related to the spirit that ebbs and flows with the seasons of life, experiences, and environment. They, however, recognized the variations and differences in appreciating this concept describing being dogmatic about definitions and traditions of health among communities as a barrier to enhancing wellness. 2) Reliance on Elders’ wisdom, skills and stories were described as facilitators for enhancing wellness.	Youth expressed a revitalized understanding of health and wellness, describing how they better understood that Indigenous health is not intricately connected to their identities through traditional activities and land-based teachings. They described a renewed outlook on wellbeing founded on Cree thought and consciousness.	None mentioned
7	Goodman et al., 2019 [76].	To explore urban Indigenous youth perspectives of health and social support.	Eagle Urban Transition Centre (EUTC), Manitoba, Canada.	Qualitative study. Recruited 18 youth between ages 15 to 24 years.	The study used photovoice within a community-based participatory research framework. Data were collected using photographs taken by youth with narratives describing 1) types and sources of social support; 2) challenges and opportunities for good health; and 3) community strengths and concerns. Follow-up interviews were also carried out.	Youth were engaged in sharing circles where they learned about photovoice and how to use photographs to facilitate dialogue. Youth were also involved in data collection, group analysis of photographs and joint interpretation of study findings.	Youth voiced several barriers and challenges to building health supports, which included. 1) Lack of trust leading to, a) no sense of belonging b) impacts in social and emotional learning c) racism and social exclusion d)hesitancy to receive support when offered and, e) low view of self-identity and self-worth (or self-esteem) and, f) social instability. 2) Systemic racism exacerbated challenges Indigenous youth encountered in building positive support networks. This ultimately resulted in youth engaging in risky behaviors (e.g., substance use, gang activity, etc.) as a way of seeking support from their peers. Facilitators to health-promoting social support among Indigenous youth included. 1) Access to activities or spaces that provide health supports (e.g., community parks, community centers, drop-in centers) 2) Peer mentorship, and 3) Cultural practices (e.g., powwow and other traditional activities that promote cultural pride and stability).	The youth acknowledged the empowerment and sense of ownership they felt by engaging in this photovoice project. One youth described how this project brought her a feeling of stability and balance. Another youth quoted, *“It’s like a sense of pride when you are learning about it, so it contributes to your self-esteem*” (page 39), referencing the Seven Grandfather Teachings (i.e., love, respect, courage, honesty, wisdom, humility, and truth) as integrated pillars of youth wellness promotion.	The authors identified the engagement of a unique group of youth experiencing challenges and receiving external community supports to which other youth may not have access as a study limitation.
8	Gray & Cote 2019 [16].	To assess whether cultural connectedness has a specific protective effect on mental health among the descendants of Indian Residential School (IRS) survivors	Anishinaabe (Algonquin) community, Canada.	Quantitative study. Random sampling of 147 community youth between 18 to 25 years	Cross-sectional study design. Data were collected using structured surveys. Key survey questions included age, gender, mental and physical health, cultural connectedness, and residential school attendance by parents and grandparents. Linear regression analysis was used to address study objectives.	Nothing regarding youth engagement was mentioned in this study.	The study showed the following results as facilitators to wellness. They include: 1) A high degree of cultural connectedness may help reverse these negative effects among youth with a family history of IRS. 2) Ways to enhance cultural connectedness included engagement with Indigenous traditions and spirituality, relationships with family/elders and positive social connections.	The findings in this study support the Indigenous notion of ‘culture as a treatment’ as Indigenous culture enhances one’s sense of meaning and self-worth and provides skills for coping with stressful circumstances.	Limitations in this study included. 1) Limitations to study design further to investigate in-depth associations between cultural connectedness and health. 2) Study used a novel single-item self-rated measure of cultural connectedness that left the concept undefined, hence open to other interpretations by respondents.
9	Hatala et al., 2019 [77]	To explore and describe how urban Indigenous youth construct a contemporary sense of themselves in miyo-wicehtowin (having good relations) with culturally grounded land-based approaches to health and wellness	Community Engagement Office, Saskatoon, Saskatchewan, Canada.	Qualitative study engaging 28 Indigenous urban youth between 15 and 24 years.	Integrative participatory research approach combining Indigenous methodologies and modified grounded theory following a two-eyed seeing framework. Data were collected using storytelling interviews that aligned with Indigenous worldviews that honor orality as a means of transmitting knowledge.	Youth were part of the Community Advisory Research Committee (CARC) and Elders, parents, and Indigenous community members. Youth were engaged as co-researchers in all phases of the research, creating an empowering space where youth could choose how and what data were collected, what parts of their stories were shared, and the ways their stories were utilized to support the research objectives.	Barriers to enhancing wellness identified in this study included; 1) Negating Indigenous peoples’ connections to land and nature in urban spaces while naturalizing rural home communities alone as potential health and wellness sources. 2) Notions that youth must maintain connections to rural homelands to maintain authentic Indigenous cultural identities associated with health and wellness advantages	Youth affirmed from this study that m*iyo-wicehtowin* (possessing good relations) with the land and nature promote health, wellness, and resilience among Indigenous youth. This was explained in the following ways: 1)*Contesting boundaries and re-locating place*: Youth re-imagine land and nature as geographically unbounded, spread out and diffused across different spaces where human activities occur. 2) Hugging trees as a kind of familial love and reciprocity 3) Gift-giving in reciprocity for the gift’s nature gives humans 4) Story-making from land-based teachings for life. 5) Regulating emotions through embodied experiences with soothing places in nature.	The authors argued that the uptake of culturally grounded land-based approaches to health and wellness among Indigenous communities should focus on rural spaces and broaden and strengthen within urban contexts for Indigenous youth in further studies.
10	Halata & Bird-Naytowhow 2020 [82].	To understand Indigenous youth experiences and journeys toward wellness in urban settings.	Gordon Tootoosis Nik̄an̄iw̄in Theatre (GTNT) in Saskatoon, Saskatchewan, Canada	A qualitative study involving 8 Cree and 2 Métis urban youth between 16 to 24 years.	Used performative theory Congruent with Etuaptmumk or “two-eyed seeing,” local Indigenous protocols, relational accountability, and cultural components (e.g., smudging, and traditional prayer offered at the start of interviews to foster an ethical, safe space). Data were collected using in-depth interviews.	Youth were engaged in a theatre program entitled ‘Circle of Voices (COV)’ at GTNT, where they learned, practiced, and performed the comedic play *Pimˆatisiwin (to celebrate life)*. They were also engaged in cultural activities as part of the workshops during this theatre program.	Facilitators described as central to enhancing wellness included: 1) *Taking responsibility:* The youth reported that through taking responsibility for one’s own personal healing and growth, they could better attain *mino-pimˆatisiwin (wellness)*. On a practical level, the youth exemplified this to actions like rising and retiring with the sun and being in flow with the seasons, being in good relationships with the environment, land, nature, and the spiritual forms that govern and operate within them. 2) *Upholding cultural values*: This was described as central to *mino-pimˆatisiwin*. Examples included sharing life experiences, respect for relationships, good moral conduct, connections to the land and nature, role modelling, spiritual practice and participation in the ceremony, and conscious awareness of the medicine wheel and its notions of wholeness, balance, harmony, growth, and healing. A barrier mentioned was the challenge in describing the performance as a lived and embodied human experience to urban inner-city Cree and Métis youth who may not understand its deep-rooted cultural significance.	1) The youth reported that engaging with culture and ceremonies was a new experience for them and thus, opened them up to other ways of being and living shaped by spiritual worlds and values. 2) the youth indicated that the engagements with Elders and GTNT staff and self-representation in the COV program helped them to reflect on their responsibility to grow and heal as individuals. 3) The authors also indicated that rehearsing in the COV program and later performing *Pimˆatisiwin* was a powerful way to build capacity at the social performance of being an Indigenous young person in inner-city Saskatoon.	The authors reported that future studies should look at how performing *pimˆatisiwin* impacts those “off-stage” in the audience and how it plays supportive roles in the lives of Indigenous young people.
11	Hutt-MacLeod et al., 2019 [78].	To describe the implementation of the ACCESS OM (AOM) objectives for youth mental health service transformation within a pre-existing Fish Net Model of transformative youth mental healthcare service.	First Nation community of Eskasoni, Nova Scotia, Canada.	Implementation and evaluation project carried out in the community with a population over 4500. More than 50% of the population was < 25 years.	Employed the two-eye seeing approach where both Indigenous and Western-influenced methods of wellness and treatment were honored and implemented. Data were collected using structured questionnaires	Youth were engaged in the design of the AOM Youth Space. A local youth council was created to guide programming, activities and services provided in this Youth Space. The youth council was also responsible for the continuing evaluation of ongoing services in the space.	Barriers identified in the report included, 1) Sustainability issues, 2) Funding, 3) scale-up and capacity of service providers to meet the demands of the community.	The AOM implementation oversaw the renovation and revitalization of the Youth Space, which served as a central location for youth to engage in activities that facilitated early identification of youth mental health needs, which informed rapid access to care. Youth were also given a choice between Western mental health services or Indigenous methods or a combination of both.	None mentioned
12	Lines et al., 2019 [79].	To explore concepts of health and healthy communities through the eyes of Indigenous youth	Yellowknife Dene First Nation (YKDFN), Northwest Territories, Canada	Mixed methods study involving 15 youth between the ages of 13 to 18 years.	Community-based participatory research (CBPR) methodology through an Indigenous research lens. Authors authentically engaged with 15 YKDFN youth and the YKDFN Wellness Division. Data were collected using sharing circles, observations, fieldnotes, Photovoice, mural art, and two quantitative surveys.	Youth were actively engaged throughout all the processes of this research. They also participated as equal partners in cultural camps, leadership workshops, photovoice, storytelling and research planning preparations.	Youth identified the following facilitators as imperatives to enhancing wellness. 1) relationship/connection to the land 2) practicing cultural skills, 3) Elders passing on traditional knowledge, 4) surviving off the land.	Youth health was enhanced by self-discovery and empowerment as youth appreciated that the symbiotic relationship between the land, environment, and people is fundamental to building a healthy community. The youth also expressed a sense of empowerment as they understood their roles in influencing health research and agency.	The authors framed future health research to include roles for youth and an on-the-land component that builds YKDFN culture, community relations, and traditional knowledge transfer.
13	Loebach et al., 2019 [80].	To explore and describe how visual technology can be used to enhance wellness for and by Indigenous youth.	Mi’kmaq Confederacy of Prince Edward Island (PEI), Canada.	Qualitative study. 11 Indigenous youth between 13 and 19 years of age.	Critical Indigenous research methods integrated with a participatory videography framework. Data were collected using digital storytelling, visual media, interviews, reflections, and field notes.	Youth were engaged in every aspect of the process as co-researchers. Activities included training workshops on video editing, software use, digital media, discussions on health and wellness, storyboarding, visual storytelling, data analyses and interpretation of short films.	Youth described the challenges of technology use to health as follows. 1) addictiveness to games and social networking platforms 2) Cyberbullying 3) Loneliness/separation from the community Youth described in their reflections how the use of technology had facilitated wellness in the following ways. 1) Building community, 2) Sharing stories 3) creating trust 4) learning about Indigenous traditions and culture.	Three short films were produced by the end of this project. In these films, youth reflect on their wellness journeys describing how this project helped self and cultural identity, empowerment, community, and healing. Engaging youth in an authentic and culturally sensitive participatory approach allowed them to express themselves in ways they didn’t think they had the capacity for. Their videos uploaded on YouTube have opened avenues for other youth to identify with the community’s struggles and change.	None mentioned.
14	Lopresti et al., 2021 [5].	To describe the key characteristics of Indigenous Youth Mentorship Program (IYMP) implementation as perceived by peer youth mentors and young adult health leaders (YAHLs).	IYMP school communities in Alberta, Canada	Qualitative study. 20 Indigenous youth between 13 to 18 years.	Ethnography. Data were collected using onsite observations, focus group discussions and semi-structured individual interviews.	The Indigenous Youth Mentorship Program (IYMP) is a peer-led health promotion program developed for elementary school children in Canada to empower Indigenous youth and reduce risk factors associated with obesity and Type 2 diabetes. Youth already participating in the IYMP program were invited for interviews and focus groups.	Five characteristics were identified as necessary for IYMP delivery. They also identify as facilitators of wellness. They included: 1) a sense of ownership by those delivering the program, 2) inclusion of Indigenous Elders/knowledge keepers, 3) establishing trusting relationships, 4) adequate program supports 5) national gatherings and shared decision making between academic and community partners	Youth participants identified positive health outcomes when the five characteristics identified in the study were upheld in the Indigenous Youth Mentorship Program.	Limitations in this study included 1) Small sample size for interviews (*n* = 4) 2) Some Indigenous youth were wary of participating in the research due to their experiences of colonial injustice and maltreatment. 3) Youth who did not feel comfortable expressing views in a group setting chose to not participate in the focus group interviews
15	Lys et al., 2018 [73].	To describe and evaluate body mapping as an arts-based activity within Fostering Open eXpression Among Youth (FOXY) among Indigenous young females	Middle to high schools in 6 Indigenous communities in Northwest Territories, Canada.	A qualitative study with 41 female Indigenous youth (aged 13 to 17 years) attended one or more FOXY workshops.	The study used a developmental evaluation methodology to produce context-specific understandings that inform ongoing innovation, support changes in direction based on feedback and emerging data and position the researcher as an integrated member of the collaborative team. Data were triangulated from four sources: body maps, semi-structured interviews, written reflections, and descriptive field notes.	FOXY is a nonprofit organization that utilizes a peer education model and employs adolescent peer leaders to cofacilitate workshops with adult facilitators. Youth were engaged in FOXY workshop activities such as visual word maps and lessons related to sexual health topics, group discussions on health, non-verbal and verbal role-playing exercises, charades, and body mapping.	The youth described body mapping as a valid data collection tool that advanced and promoted trust and youth voices in research while reducing verbal communication barriers and facilitating the collection of rich data on Indigenous youth experiences	Youth affirmed that using the body mapping in FOXY encouraged and supported wellness and healing through self-reflection, introspection leading to self-discovery, and personal/cultural connectedness while enhancing the ability to process difficult emotions.	The authors reported that directions for future research could explore repeat exposure to body mapping interventions and their impact on well-being
16	Merati et al., 2020 [8].	To explore how Cree youth perceived youth health and youth engagement in health and health planning.	Eeyou Istchee territory of northern Quebec, Canada	Qualitative descriptive study recruiting ten Cree youth aged between 10 to < 25 years.	The study adopted a community-based participatory research approach. Data were collected using focus group discussions and key-informant interviews	Youth were engaged in 3 levels on an engagement spectrum. The first level was defined as participation where youth partook in cultural activities and ceremonial events. The second was described as youth council membership, where youth were nominated to represent the youth voice and shared decision-making. The third level of engagement was defined as planners were decision-makers and advocated for planning and action.	Youth identified barriers to youth engagement in leadership capacities in the following, 1) Adults not taking their voices seriously, 2) Micro-managing or terminating youth programs due to low attendance.	Wellness was facilitated through youth engagement in these three levels. Youth described how they needed to be continually engaged to be healthy and healthy to be involved.	Limitations identified in the study included. 1) Small sample size: the study focused on a limited number of Cree youth. 2) Limited tie for meaningful engagement: engagement with youth using the CBPR lasted eight days.
17	Njeze et al., 2020 [83].	To examine and describe intersecting individual and social factors that enhance resilience and wellness among Indigenous urban residing youth.	Community Engagement Office, Saskatoon, Saskatchewan, Canada.	Qualitative study engaging 6 Indigenous urban youth between 15 and 24 years.	Case study design using an integrative framework of intersectionality theory and resilience while drawing on Indigenous methodologies, a “two-eyed seeing” approach, and Stake’s case study methodology involving multiple data sources (i.e., sharing circles, conversational interviews, photovoice and naturalistic interactions).	Youth were engaged as co-researchers in all phases of the research and were actively involved as members of the Community Advisory Research Committee (CARC) guiding the research processes.	The study concluded that several individual and sociocultural processes intersect and contribute to resilience among Indigenous youth. These processes were described as facilitators, which included; 1) Resilience (wellness) is enhanced when grounded at a cultural level and focused on community, spirituality, and cultural relationships. 2) Resilience (wellness) is enhanced with a strong will and determination to acquire and attain lifelong development and lead exemplary lives that positively influence other Indigenous youth. 3) Resilience (wellness) is enhanced by creating safe spaces for Indigenous youth to escape, recharge and return revitalized.	Urban residing Indigenous youth described how they struggled with various forms of acute hardships from intersecting individual, social and cultural lines in 6 case study vignettes. They, however, told how resilience (wellness) was enhanced in their stories summarized in the following ways; 1) By building and strengthening their cultural identity and family connections 2) By engaging in social and cultural groups providing services to themselves and their immediate communities. 3) By keeping a positive outlook on life through arts practices (music, dances, singing, painting, etc.).	Limitations reported included; 1) Intrinsic limitations of using intersectionality theory do not include all possible factors or processes. 2) Failing to engage voices of family members of participating youth to aid triangulation. Areas for further research should continue to explore and advance the intersectionality of resilience framework to fully understand how different social processes intersect and support youth resilience and wellness in unique ways.
18	Plazas et al., 2019 [81].	To engage Indigenous youth in popular theatre to explore inequities in access to health services for Indigenous people.	Community schools in Alberta Indigenous territory, Canada.	Qualitative study. Age bracket and the number of youth participants not mentioned.	Ethnographic study design integrated with Paulo Freire’s pedagogical work on critical consciousness and reflexivity. Data were collected by observation, interviews, focus groups, story-sharing, real-life vignettes, and field notes.	Popular theatre uses various theatre games and exercises to help build community and communication skills and deepen understanding of oneself and others. Youth were engaged in theatre workshops, rehearsals, games, exercises, story sharing and discussions that evoked critical thinking and consciousness	At the end of the theatre program, the youth produced a 1-hour production of 5 skits with characters, plots, and storylines that described the processes that continued to challenge or facilitate personal transformation and resilience concerning wellness. Facilitators: 1) dialogues that engage critical thinking and mutual learning 2) community support and leadership 3) self-motivation is driven by cultural self-awareness and self-appraisal. Barriers 1) misperception of a lack of control for self-governance in Indigenous communities	Youth expressed that they better understood the connections between their community issues and healthcare equity by engaging in this form of interactive theatre. They expressed a feeling of self-awareness and the capacity to thrive in their communities.	Authors identified that the lack of action following the interactive performances was a limitation and direction for further research inquiry
19	Saini et al., 2020 [84].	To share public health information about acute gastrointestinal illness (AGI) by co-developing a whiteboard video with Inuit youth, community members and government partners and evaluating its efficacy.	Rigolet Inuit community, Nunatsiavut, Newfoundland and Labrador, Canada	Qualitative study. Six youth between 11 to 12 years engaged in the co-production. Fifty-four community members were interviewed during the evaluation.	The study used an integrated participatory action research and evaluation framework. A short-term evaluation of the community’s reaction to the whiteboard video was conducted. Data were collected through interviews and focus group discussions.	Whiteboard videos utilize visual, audio, and oral elements to communicate narratives. Rigolet youth were engaged in the co-production processes through workshops and training sessions. The youth selected all the video characters and provided feedback on proposed storyboards and storylines.	Facilitators mentioned: Engaging Rigolet youth in the video co-development process ensured community relevance of the video.	1) Wellness was enhanced by co-producing with youth a 4m46s video discussing locally identified public health messages about ways to reduce the risk of AGI. 2) Evaluation results suggested the video reinforced health knowledge and encouraged behavioral change. 3) Evaluation participants believed the video promoted Inuit health because of locally relevant visuals and narrative, which reflected Inuit art and storytelling traditions.	None mentioned.
20	Sanchez-Pimienta et al., 2021 [44].	To describe insights gained from a youth co-led participatory digital storytelling project focused on Indigenous health promotion strategies.	M’Wikwedong Indigenous Friendship Centre in Owen Sound, Ontario, Canada.	Qualitative type study engaging 4 Indigenous youth aged between 17 to 22 years.	The study used an integrative framework of participatory action research and exploratory case study design. Data were collected using group interviews and discussion circles with youth and the research team.	Digital storytelling is a facilitated technique that guides participants in identifying an idea and transforming it into a compelling story presented in short audio-visual formats. Youth were engaged in mentorship and training sessions on media gathering and video editing	Facilitators identified: The following were identified as the ‘ethos of connection’ that underpinned Indigenous health promotion, thus, facilitated wellness. They included a) restoring balance in relationships, b) Indigenous youth guided leaderships, and c) egalitarianism and inclusiveness of knowledge from Western ways of knowing (that proposed a notion of control over Indigenous knowledge)	1) Youth researchers created four videos describing their understanding of health and health promotion from an Indigenous perspective. These videos represented themes that described ways the M’Wikwedong Centre reinforced connections to youth, their sense of self, place in the city and Indigenous cultures. Youth documented in these videos how they found healing, self-identity, and balance through the research processes.	None mentioned.

### Synthesis of results

**Fig. 2:**
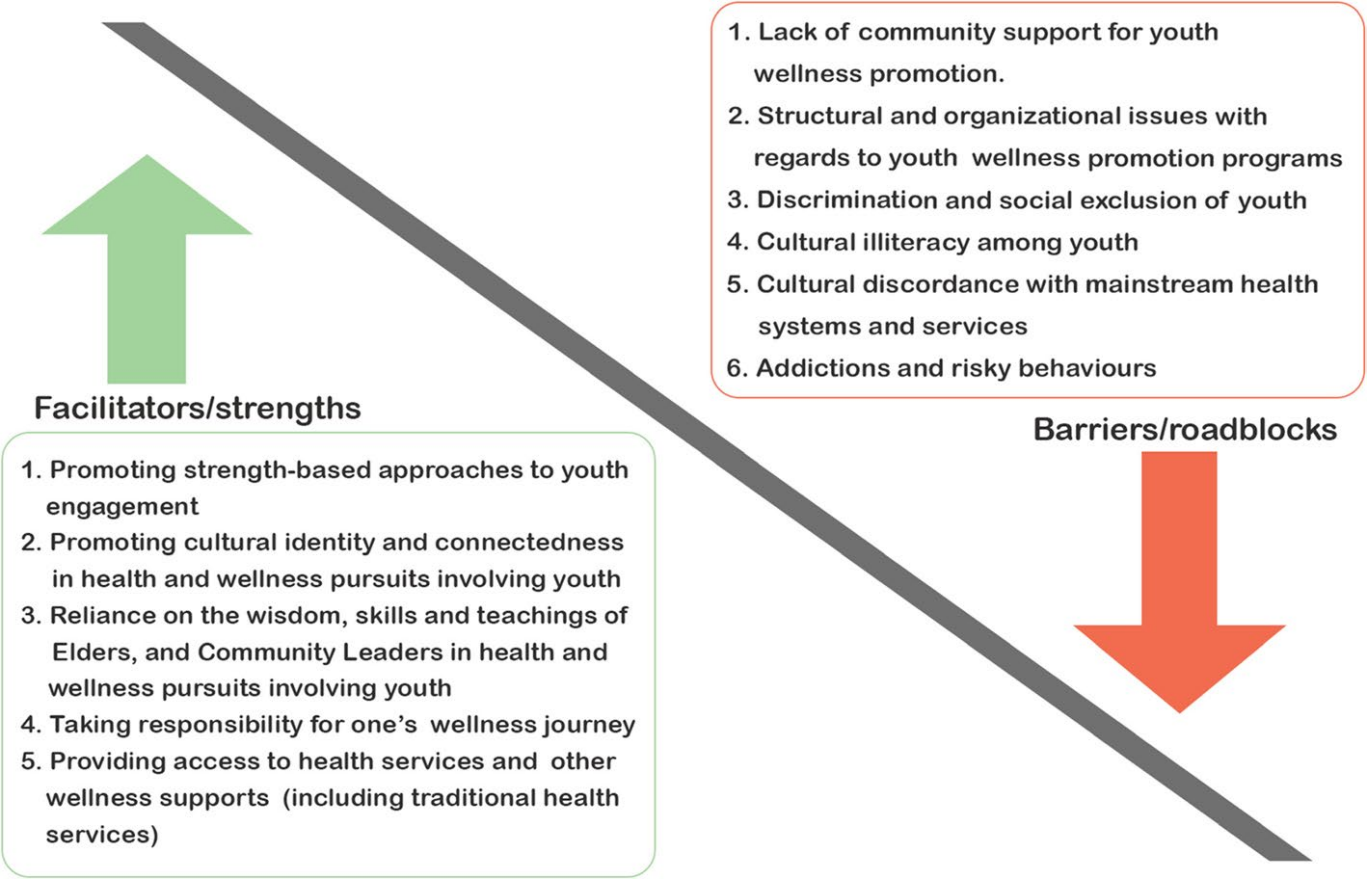
Summary of facilitators/strengths and barriers/roadblocks to enhancing wellness by, for and with Indigenous youth

The key *facilitators/strengths* and *barriers/roadblocks* to enhancing health and wellness by, for, and with Indigenous youth that emerged from the included studies are described in Table 6, in descending order of major themes for the frequency of citation by included articles per theme. The facilitators/strengths and barriers/roadblocks have also been categorized into sub-themes under five major themes for facilitators/strengths and six major themes for barriers/roadblocks. Health outcomes/programs examined by included studies included suicide prevention [40], mental health promotion [71, 74], HIV prevention [75], wellness promotion through youth empowerment and cultural activism [5, 8, 16, 57, 72,,76, 77, 78,79, 80], social health [76, 83], land-based healing and wellness [77, 82], art-media based therapy and wellness [44, 73, 81, 84]. An overview of the facilitators/strengths and barriers/roadblocks to enhancing health and wellness by, for, and with Indigenous youth is presented in Fig. 2.

**Table 6:** Key themes identified as facilitators/strengths and barriers/roadblocks to enhancing health and wellness by and with Indigenous youth in Canada [*n* = 20]

	Facilitators	Freq (%)^a^	Article citations		Barriers	Freq (%)^a^	Article citations
**1.**	**Promoting strength-based approaches to youth engagement**	19 (95.0)	[5, 8, 16, 44, 57, 71–84]	**1.**	**Lack of community support (social, financial, organizational for wellness promotion among Indigenous youth)**	11 (55.0)	[5, 44, 57, 72, 74–78, 80, 81]
1a	Peer-to-peer mentoring (Indigenous youth mentored by, and with other Indigenous youth)	15 (75.0)	[5, 8, 44, 57, 71, 73–76, 79–84]	**2.**	**Structural and organizational issues within Indigenous communities with regards to wellness promotion programs**	10 (50.0)	[5, 8, 72, 73, 76–78, 81–83]
1b	Engaging youth in activities that develop and promote self-determination, capacity budling, and empowerment (e.g., guided storyboarding, performative art, arts-based therapy, etc.)	15 (75.0)	[5, 8, 44, 57, 72–74, 76–80, 82–84]	2a	Community concerns affecting the sustainability of instituted wellness programs/strategies	4 (20.0)	[5, 8, 78, 81]
1c	Building positive relationships with one another, with nature and the environment	14 (70.0)	[5, 8, 44, 57, 72, 73, 76, 77, 79–84]	2b	Dogmatism about definitions regarding traditions of health among Indigenous communities	4 (20.0)	[72, 77, 82, 83]
1d	Showing kindness to one another	9 (45.0)	[5, 16, 44, 57, 77, 79–81, 83]	2c	Social and structural instability within communities	3 (15.0)	[8, 76, 83]
1e	Engaging youth in discussions/conversations to stimulate critical consciousness, mutual learning, and transformative change.	6 (30.0)	[5, 8, 75, 76, 79, 81]	2d	Low capacity of service providers (e.g., vendors, health service centers, social service centers, etc.) to meet the demands of communities.	3 (15.0)	[73, 78, 81]
1f	Engaging youth in traditional sporting activities	4 (20.0)	[57, 76, 82, 83]	2e	Misperception of a lack of control for self-governance in Indigenous communities	1 (5.0)	[81]
**2.**	**Promoting cultural identity and connectedness by engaging youth in cultural activities during health and wellness programs or pursuits**	16 (80.0)	[8, 16, 40, 44, 57, 71, 72, 75–77, 79–84]	**3.**	**Discrimination and social exclusion of Indigenous youth**	8 (40.0)	[5, 8, 44, 57, 74, 76, 80, 83]
**3.**	**Reliance on Elders’ or Knowledge Keepers’ (and Community leaders) wisdom, skills, and teachings in pursuing health and wellness programs by, for and with Indigenous youth**	10 (50.0)	[5, 16, 44, 72, 77, 79–81, 83, 84]	3a	Racism (personal, interpersonal, structural, and systemic racism)	5 (25.0)	[5, 8, 76, 80, 83]
**4.**	**Taking personal responsibility for one’s wellness journey**	8 (40.0)	[44, 57, 72, 74, 79, 80, 82, 83]	3b	Low view of self and self-identity (i.e., self-deprecation and self-exclusion from engaging in youth activities)	5 (25.0)	[8, 44, 76, 80, 83]
**5.**	**Providing access to health services and other wellness supports (including traditional health services)**	2 (10.0)	[76, 78]	3c	Mental health stigmatization (personal, interpersonal, structural, and systemic racism)	3 (15.0)	[73, 74, 76]
				3d	Lack of inclusivity of Indigenous traditional activities into Canadian teaching institutions	2 (10.0)	[76, 77]
				3e	Bullying, hunger and abuse	2 (10.0)	[57, 80]
				**4.**	**Cultural illiteracy among Indigenous youth**	7 (35.0)	[44, 57, 73–75, 83, 84]
				**5.**	**Cultural discordance with mainstream health systems and services**	6 (30.0)	[5, 8, 43, 44, 74, 76]
				**5a**	Discordance between Western and Indigenous approaches to health and wellness.	4 (20.0)	[8, 43, 44, 76]
				5b	Mistrust of mainstream health services leading to hesitancy to receive support when offered	3 (15.0)	[5, 74, 76]
				**6.**	**Addictions and risky behaviors (e.g., substance use, gang activity, etc.)**	5 (25.0)	[44, 57, 75, 76, 80]

*Freq* Frequency

^a^Frequency is the number of cited articles per theme. Percentages are out of the 20 articles included in the review. Bold font denotes main themes

### Facilitators/strengths to enhancing health and wellness by, for, and with indigenous youth

Five major themes emerged and were identified as facilitators/strengths to enhancing health and wellness by, for, and with Indigenous youth in Canada. The most identified facilitator/strength of health and wellness among Indigenous youth in Canada, identified in 19 [95%] of the included studies, was the promotion of strength-based approaches to engaging with youth in the community [5, 8, 16, 44, 57, 71–84]. A number of sub-themes also emerged from this major theme to include: peer-mentoring [5, 8, 44, 57, 71, 73–76, 79–84]; engaging youth in programs that developed and promoted self-determination, capacity building and empowerment [5, 8, 44, 57, 72–74, 76–80, 82–84]; building positive relationships and social connections with others, nature and the environment [5, 8, 44, 57, 72, 73, 76, 77, 79–84]; showing kindness to one another [5, 16, 44, 57, 77, 79–81, 83]; and engaging youth in cultural activities [57, 76, 82, 83] that stimulate or encourage mutual learning, enhance critical consciousness and cause transformative change [5, 8, 75, 76, 79, 81]. The next most common facilitator identified in 16 [80%] of included studies was enhancing cultural identity and connectedness through youth engagement in cultural activities [8, 16, 40, 44, 57, 71, 72, 75–77, 79–84]. Other facilitators included: reliance on the wisdom, skills, and teachings of community Elders, Traditional Knowledge Keepers and community leaders in the pursuit of health and wellness promotion with Indigenous youth [5, 16, 44, 72, 77, 79–81, 83, 84]; taking responsibility for one’s journey to wellness [44, 57, 72, 74, 79, 80, 82, 83]; and providing access to health services and other wellness supports (including traditional health services) for youth in Indigenous communities [76, 78]. A summary of the facilitators/strengths is provided in Fig. 2.

### Barriers/roadblocks to enhancing health and wellness by, for, and with indigenous youth

Six major themes emerged and identified as barriers/roadblocks to enhancing health and wellness by, for and with Indigenous youth in Canada. The most identified barrier/roadblock to enhancing health and wellness identified in 55% (11/20) of the included articles was a lack of community support [including social, financial, and organizational support] for wellness promotion strategies among Indigenous youth [5, 44, 57, 72, 74–78, 80, 81]. Structural and organizational issues within Indigenous communities regarding wellness promotion strategies were identified as the second most common barrier/roadblock to enhancing wellness in 50% [10/20] of included studies [5, 8, 72, 73, 76–78, 81–83]. These structural and organizational issues included: Indigenous community problems or concerns affecting the sustainability of instituted wellness programs/strategies [5, 8, 78, 81]; dogmatism and debates about definitions regarding traditions of health among Indigenous communities [72, 77, 82, 83]; social and structural instability within communities (e.g., leadership concerns) [8, 76, 83]; modest to low capacity of service providers (e.g. vendors, health service centers, social service centers, etc.) to meet the demands of communities [73, 78, 81]; and the misperception of a lack of control for self-governance in Indigenous communities [81]. Discrimination and social exclusion of Indigenous youth were also identified as a barrier/roadblock to enhancing wellness in eight (40%) studies included [5, 8, 44, 57, 74, 76, 80, 83]. Forms of discrimination and social exclusion identified as subthemes included: Racism (e.g., personal, interpersonal, structural and systemic racism) [5, 8, 76, 80, 83]; low self-esteem and a low view of self-identity leading to self-deprecation and self-exclusion from engaging in youth activities [8, 44, 76, 80, 83]; mental health stigmatization [73, 74, 76]; lack of inclusivity of traditional Indigenous activities into Canadian teaching institutions [76, 77]; and all forms of bullying, abuse and hunger [57, 80]. Other barriers/roadblocks included: cultural illiteracy among Indigenous youth [44, 57, 73–75, 83, 84]; friction between Western and Traditional methods of promoting health and wellness [5, 74, 76, 77]; and risky behaviours such as gang activity, substance use/abuse and addictions [44, 57, 75, 76, 80]. A summary of the barriers/roadblocks is provided in Fig. 2.

## Discussion

Scoping reviews determine the extent, range, and quality of evidence on any chosen topic [60–63]. In addition, they can be used to map and describe what is known about an identified topic to identify existing gaps in the literature regarding the chosen topic [60–63]. In this scoping review, the peer-reviewed evidence regarding facilitators/strengths and barriers/roadblocks to enhancing health and wellness by, for and with Indigenous youth in Canada were mapped and synthesized. Key facilitators/strengths highlighted included: promoting culturally appropriate interventions [8, 16, 40, 44, 57, 71, 72, 75–77, 79–84] using strength-based approaches [5, 8, 16, 44, 57, 71–84]. Key barriers to enhancing health and wellness by, for and with Indigenous youth identified in this review were the lack of community support for wellness promotion activities among Indigenous youth [5, 44, 57, 72, 74–78, 80, 81] and structural/organizational issues within Indigenous communities [5, 8, 72, 73, 76–78, 81–83].

Strength-based approaches empower community members, academic researchers, and policymakers to effect community change while focusing on what has worked in the past and the community vision for success in the future [79]. This is contrasted with the common narrative in most studies exploring Indigenous health and wellness that focused on why and where the community has failed to thrive [79]. Promoting strength-based interventions by, for, and with Indigenous youth works in parallel with ensuring that health interventions are culturally appropriate [44, 79] because Indigenous epistemologies or ways of knowing see reality as intricate processes of interdependent relationships between humans, nature, and the spirit world [44, 77]. As such, wellness promotion in Indigenous communities should emphasize support for their traditional values such as respect, trust, non-judgement, and relationality, all of which support cultural revitalization [26, 71].

Conversely, wellness promotion in Indigenous communities should disavow the use of Western-based epistemologies that embrace and emphasize control over risk factors and health [44, 79]. The definition and perception of health and wellness by Indigenous peoples are starkly different from the Western perspective of health promotion [44, 79] which was found in our study to be a barrier/roadblock to enhancing health and wellness by, for and with Indigenous youth [8, 43, 44, 76]. Because of these contrasting and conflicting views on health and wellness, research carried out with Indigenous communities *must* be grounded in their culture. Elder Jim Dumont – a professor of Native Studies and a member of the Shawanaga First Nation on Eastern Georgian Bay, when describing the role of Indigenous culture in facilitating wellness among Indigenous peoples, defined Indigenous culture as a “*facilitator to spiritual expression”* [85 p.11]. He described Indigenous culture as “an expression of the life-ways, the spiritual, psychological, social, and material practice of the Indigenous worldview, which attends to the whole person’s spiritual desire to live life to the fullest” [85 p.9]. This was the way of life for Indigenous peoples before colonization [2]. Back then, Indigenous peoples honoured and utilized traditional methods and practices connected to their respective unceded homelands to promote and sustain health and wellness by themselves within their respective communities [2, 16, 86]. These cultural practices provided and promoted health and wellness for the community, the peoples, the lands, and the environment [2].

Furthermore, Indigenous wellness promotion by, for and with Indigenous youth should go beyond making mainstream health promotion strategies more culturally appropriate. Indigenous wellness promotion should also invite youth as partners and co-researchers to authentically engage with the community, acknowledging their needs while working together *with them* to identify opportunities for change (which should include shared power and responsibilities in the relationship dynamic). This must be the fundamental principle for any work done by, for, or with Indigenous communities (i.e., authentic engagement) [54, 55, 59]. Authentic engagement is working and walking *with* rather than *on* communities [54] in a way that encourages respectful, compassionate, and genuine interest in the work undertaken by all partners involved [54, 55, 57, 87, 88]. In authentically engaging with Indigenous communities, emphasis should be placed on *connecting with*, rather than *controlling,* community members [44, 89]. By doing so, enhances a community’s ability to answer their issues by identifying their community strengths and assets, considering opportunities for change, and co-creating meaningful solutions to mitigate them.

The Tri-Council Policy Statement (TCPS) on Ethical Conduct for Research involving Humans indicates in Chapter 9 that, where research involves First Nations, Métis, and Inuit peoples and their communities, they are to have a role in shaping and co-creating research that affects them; with respect being given to the autonomy of these communities and the individuals within them to decide to participate [90]. Our study showed that where youth were engaged as partners and co-researchers, promoted self-determination, capacity building and ultimately enhanced wellness [8, 40, 44, 57, 72, 74–77, 79, 84].

From the outcomes of this review, youth were engaged as partners or co-researcher in 55% of the included articles using research approaches such as community-based participatory research [CBPR], photovoice, visual voice, participatory videography, performative arts, participatory narrative, and storytelling methods [8, 40, 44, 57, 72, 74–77, 79, 84]. This review demonstrated that these methods helped foster an environment for transformative learning, reciprocal transfer of expertise, shared decision-making, and co-ownership of the research processes [8, 40, 44, 57, 72, 74–77, 79, 84]. For example, Goodman et al. identified that through photovoice, youth identified how racism negatively influenced the types of social supports and relationships formed in their community, leading to improved access to mental health-promoting social programs [76]. Anang et al. reported that engaging Indigenous youth as co-researchers in exploring ways to promote suicide prevention revitalized awareness of their cultural identity, which was identified as a protective factor to youth suicide [40]. A group of First Nation girls involved in the Girl Power Program designed to build and foster empowerment using youth participatory action research approach indicated that working as co-researchers/co-creators in the program empowered them to find healing from wounded spirits, which helped enhance positive changes towards wellness through *āhkamēyimowin* (perseverance) [57]. Thus, we can conclude from our study that engaging youth as partners in research processes optimizes their personal experiences and gives them a voice which can stimulate action.

Engaging Indigenous youth in the co-creation of wellness strategies should also involve community Elders, Traditional Knowledge Keepers, and other Indigenous community leaders. This review demonstrated that reliance on the wisdom of Elders, Traditional Knowledge Keepers and Indigenous community leaders facilitated and enhanced wellness among Indigenous youth [5, 16, 44, 72, 77, 79–81, 83, 84, 91]. Elders, Traditional Knowledge Keepers, and Indigenous community leaders play a central role in increasing awareness related to the community’s histories, languages, knowledge, and ways of knowing [91, 92]. For non-Indigenous researchers and allies, Elders and Traditional Knowledge Keepers can provide formal and informal teachings on: histories of the Indigenous community in question, their world views, languages in the community, arts, crafts and songs, value systems in the nation/community; knowledge of traditional plants and medicines; clan teachings in the nation/community; ceremonial knowledge or protocols; and understanding of wellness in the community that can increase cultural awareness and build Indigenous research competencies for non-Indigenous researchers and allies [91–93]. Hence, engaging Elders, Knowledge Keepers and Indigenous community leaders in youth wellness programs can provide an avenue for mutual learning, guiding non-Indigenous researchers/allies towards cultural appropriateness in co-developing youth-driven wellness strategies.

### Practical implications

Overall, this review emphasized the importance of promoting wellness among Indigenous youth using ‘*culture as strength*’ rather than imposing control measures on Indigenous values. The historical experiences of Indigenous youth have revealed traumatic and distressful pasts propagated by the cumulative intergenerational impacts of colonization which evolved from Residential Schools, Day Schools, and the Sixties Scoop [15, 16, 33, 94, 95]. The 2015 Truth and Reconciliation Commission of Canada’s 96 Calls-to-Action stressed the need to decolonize mainstream health promotion strategies and embrace the promotion of self-determination in the use of and access to traditional knowledge, therapies, and healing practices Indigenous peoples [95, 96]. This review provided a foundation for authentically engaging Indigenous youth in the co-creation of culturally appropriate wellness promotion strategies/programs driven and sustained by authentically engaged Indigenous youth in the community. Considering the number of qualitative studies we found in our review, a meta-synthesis of qualitative studies may guide future directions based on the findings in our study to further pursue to understand, appraise, summarize, and combine qualitative evidence to address the specific research questions particularly around the influences and experiences of cultural connectedness and wellness among Indigenous youth in Canada. Nonetheless, this review also contributes to the growing literature identifying strength-based approaches to enhancing health and wellness among Indigenous peoples in Canada.

### Study limitations

This review aimed to provide an entire scope of all original studies published in peer-reviewed journals to allow for as broad a scope of literature synthesis as possible. However, this study is not without limitations. First, the search was limited to multiple library databases, including the University of Saskatchewan’s Indigenous Studies Portal (iPortal) [66]. Although this review produced many peer-reviewed and original studies, there is a potential that other relevant articles and reports were missed because we did not search the grey literature. Secondly, because this review was limited to peer-reviewed articles published in English, it is possible that potentially relevant studies in other languages were omitted. Moreover, the outcomes of this review are limited to the nature of the data reported in the articles included in the review. Additionally, we acknowledge the differences and nuances in Indigenous practices, values and culture which limits the generalizability of our review findings. Lastly, some of the studies in the scoping review utilized Indigenous study designs and methods that could not be appropriately evaluated using the JBI Critical Appraisal Tools [70].

## Conclusion

This scoping review identified ways health and wellness can be enhanced by, for, and with Indigenous youth by identifying facilitators/strengths and barriers/roadblocks to enhancing health and wellness among Indigenous youth from identified studies published between January 1, 2017, and May 22, 2021. The outcomes of this review showed that promoting culturally based and appropriate interventions using strength-based approaches were key facilitators/strengths to enhancing health and wellness among Indigenous youth. Thus, the outcomes demonstrate the continued need to promote programs grounded in culture as a part of enhancing health and wellness while authentically engaging Indigenous youth in health and wellness strategies, interventions, and programs.

## Supplementary Information

**Additional file 1: Supplementary Material File 1.** PRISMA-ScR Checklist.

**Additional file 2: Supplementary Material File 2.** Summary of critical appraisals of individual sources of evidence.

**Additional file 3: Supplementary Material File 3.** List of Terminology.

## Data Availability

Data sharing is not applicable to this article as no datasets were generated or analyzed during the current study.

## Abbreviations

CBPR: Community-based participatory research
FN: First Nations
FTA: Full-Text Articles
iPortal: University of Saskatchewan’s Indigenous Studies Portal
JBI: Joanna Briggs Institute
MeSH: Medical Subject Headings
PAR: Participatory action research
PRISMA: Preferred Reporting Items for Systematic Reviews and Meta Analyses
PRISMA-ScR: Preferred Reporting Items for Systematic Reviews and Meta-Analyses extension for Scoping Reviews
