# Supplementary material for: Enhancing health and wellness by, for and with Indigenous youth in Canada: a scoping review
Source: BMC Public Health. 2022 Aug 29;22:1630. doi: 10.1186/s12889-022-14047-2 (PMC9422134; doi:10.1186/s12889-022-14047-2)
Supplement: Supplementary file 2 — Additional file 2: Supplementary Material File 2. Summary of critical appraisals of individual sources of evidence. [file 12889_2022_14047_MOESM2_ESM.docx]

**Supplementary Material File 2**

**Summary of critical appraisals of individual sources of evidence**

| **S/N** | **Qualitative Studies** | Anang et al., 2019 | Etter et al., 2019 | Goodman et al., 2019 | Halata & Bird-Naytowhow 2020 | Hatala et al., 2019 | Hutt-MacLeod et al., 2019 |
| --- | --- | --- | --- | --- | --- | --- | --- |
| 1 | Is there congruity between the stated philosophical perspective and the research methodology? | Yes | Yes | Yes | Yes | Yes | Yes |
| 2 | Is there congruity between the research methodology and the research question or objectives? | Yes | Yes | Yes | Yes | Yes | Yes |
| 3 | Is there congruity between the research methodology and the methods used to collect data? | Yes | Yes | Yes | Yes | Yes | Yes |
| 4 | Is there congruity between the research methodology and the data representation and analysis? | Yes | Yes | Yes | Yes | Yes | Yes |
| 5 | Is there congruity between the research methodology and the interpretation of the results? | Yes | Yes | Yes | Yes | Yes | Yes |
| 6 | Is there a statement locating the researcher culturally or theoretically? | Yes | No | Yes | No | No | No |
| 7 | Is the influence of the researcher on the research, and vice versa, addressed? | No | Yes | Yes | Yes | Yes | Unclear |
| 8 | Are participants and their voices adequately represented? | Yes | Yes | Yes | Yes | Yes | Yes |
| 9 | Is the research ethical according to current criteria or for recent studies, and is there evidence of ethical approval by an appropriate body? | Yes | Yes | Yes | Yes | Yes | Yes |
| 10 | Do the conclusions drawn in the research report flow from the analysis or interpretation of the data? | Yes | Yes | Yes | Yes | Yes | Yes |

Key: Y= Yes; N=No; U=Unclear; NA=Not applicable

| **S/N** | **Qualitative Studies** | Lines et al., 2019 | Lys et al., 2018 | Gaspar et al., 2019 | Gaudet & Chilton, 2018 | Loebach et al., 2019 | Lopresti et al., 2020 |
| --- | --- | --- | --- | --- | --- | --- | --- |
| 1 | Is there congruity between the stated philosophical perspective and the research methodology? | Yes | Yes | Yes | Yes | Yes | Yes |
| 2 | Is there congruity between the research methodology and the research question or objectives? | Yes | Yes | Yes | Yes | Yes | Yes |
| 3 | Is there congruity between the research methodology and the methods used to collect data? | Yes | Yes | Yes | Yes | Yes | Yes |
| 4 | Is there congruity between the research methodology and the data representation and analysis? | Yes | Yes | Yes | Yes | Yes | Yes |
| 5 | Is there congruity between the research methodology and the interpretation of the results? | Yes | Yes | Yes | Yes | Yes | Yes |
| 6 | Is there a statement locating the researcher culturally or theoretically? | Yes | Yes | Unclear | No | Yes | No |
| 7 | Is the influence of the researcher on the research, and vice versa, addressed? | Yes | Yes | No | No | Yes | Yes |
| 8 | Are participants and their voices adequately represented? | Yes | Yes | Yes | Yes | Yes | Yes |
| 9 | Is the research ethical according to current criteria or for recent studies, and is there evidence of ethical approval by an appropriate body? | Yes | Yes | Yes | Yes | Yes | Yes |
| 10 | Do the conclusions drawn in the research report flow from the analysis or interpretation of the data? | Yes | Yes | Yes | Yes | Yes | Yes |

Key: Y= Yes; N=No; U=Unclear; NA=Not applicable

| **S/N** | **Qualitative Studies** | Flicker et al., 2019 | Plazas et al., 2019 | Merati et al., 2020 | Njeze et al., 2020 | Saini et al., 2018 |
| --- | --- | --- | --- | --- | --- | --- |
| 1 | Is there congruity between the stated philosophical perspective and the research methodology? | Yes | Yes | Yes | Yes | Yes |
| 2 | Is there congruity between the research methodology and the research question or objectives? | Yes | Yes | Yes | Yes | Yes |
| 3 | Is there congruity between the research methodology and the methods used to collect data? | Yes | Yes | Yes | Yes | Yes |
| 4 | Is there congruity between the research methodology and the data representation and analysis? | Yes | Yes | Yes | Yes | Yes |
| 5 | Is there congruity between the research methodology and the interpretation of the results? | Yes | Unclear | Yes | Yes | Yes |
| 6 | Is there a statement locating the researcher culturally or theoretically? | Yes | No | Yes | Yes | Yes |
| 7 | Is the influence of the researcher on the research, and vice versa, addressed? | Yes | No | Yes | Yes | Yes |
| 8 | Are participants and their voices adequately represented? | Yes | Yes | Yes | Yes | Yes |
| 9 | Is the research ethical according to current criteria or for recent studies, and is there evidence of ethical approval by an appropriate body? | Yes | Unclear | Yes | Yes | Yes |
| 10 | Do the conclusions drawn in the research report flow from the analysis or interpretation of the data? | Yes | Yes | Yes | Yes | Yes |

Key: Y= Yes; N=No; U=Unclear; NA=Not applicable

| **S/N** | **Case studies** | Njeze et al., 2020 | Sanchez-Pimienta et al., 2020 |
| --- | --- | --- | --- |
| 1 | Were patient’s demographic characteristics clearly described? | Yes | Yes |
| 2 | Was the patient’s history clearly described and presented as a timeline? | Yes | No |
| 3 | Was the current clinical condition of the patient on presentation clearly described? | Yes | Yes |
| 4 | Were diagnostic tests or assessment methods and the results clearly described? | Yes | Yes |
| 5 | Was the intervention(s) or treatment procedure(s) clearly described? | Yes | Yes |
| 6 | Was the post-intervention clinical condition clearly described? | Yes | Yes |
| 7 | Were adverse events (harms) or unanticipated events identified and described? | Yes | No |
| 8 | Does the case report provide takeaway lessons? | Yes | Yes |

Key: Y= Yes; N=No; U=Unclear; NA=Not applicable

| **S/N** | **Cross sectional studies** | Crooks et al. 2017 | Gray & Cote 2019 |
| --- | --- | --- | --- |
| 1 | Were the inclusion criteria clearly defined? | Yes | Yes |
| 2 | Were the study subjects and setting described in detail? | Yes | Yes |
| 3 | Was the exposure measured in a valid and reliable way? | Yes | Unclear |
| 4 | Were objective, standard criteria used to measure the condition? | Yes | Yes |
| 5 | Were confounding factors identified? | Unclear | Unclear |
| 6 | Were strategies to deal with confounding factors stated? | Unclear | Unclear |
| 7 | Were the outcomes measured in a valid and reliable way? | Yes | Yes |
| 8 | Were appropriate statistical analyses used? | Yes | Yes |

Key: Y= Yes; N=No; U=Unclear; NA=Not applicable
