# Supplementary material for: Enhancing health and wellness by, for and with Indigenous youth in Canada: a scoping review
Source: BMC Public Health. 2022 Aug 29;22:1630. doi: 10.1186/s12889-022-14047-2 (PMC9422134; doi:10.1186/s12889-022-14047-2)
Supplement: Supplementary file 3 — Additional file 3: Supplementary Material File 3. List of Terminology. [file 12889_2022_14047_MOESM3_ESM.docx]

**Supplementary Material File 3**

**List of Terminology**

**Colonization:** the process of settling or establishing control over Indigenous peoples. This process can take the form of early contact and settlement but can also take the form of modern policies and legislation that continues to impose control and regulate the lives of Indigenous peoples.

**Elder:** an influential community member and knowledge holder who is recognized and respected for their teachings, values, and principles.

**First Nation:** First Nation people are the Aboriginal peoples of Canada who are located south of the Arctic. This is a term rarely used by Indigenous peoples to describe themselves, although it is also not considered offensive or problematic. It recognizes that Indigenous Peoples are distinct groups, without the political connotations. Individual nations also have terms for themselves as Indigenous people, for example, the Mi’kmaq use the term “L’nuk” or “L’nu.”

**Indigenous:** an inclusive term (replacing the term Aboriginal which is considered offensive and problematic) which is becoming increasingly popular for those who see themselves as Indigenous. It means “of the land” and it not imposed by law or the Government of Canada.

**Indigenous Peoples:** While the term Indigenous has become more commonly used around the world, it is contested by some because it does not acknowledge the unique identities or distinct rights of First Nations, Inuit and Métis peoples. Always refer to the specific group (First Nations, Inuit or Métis) rather than generalizing with a collective phrase like Indigenous.

**Inuit:** a distinct group of Aboriginal Canadians who are neither First Nations, or Métis. In Inuktitut, Inuit means “the People.” Historically the term Eskimo was used to describe the Indigenous peoples of Alaska, Canada, and Greenland; however, Eskimo is seen as demeaning and unacceptable, and has fallen out of use when referring to Arctic and subarctic peoples.

**Knowledge Keeper:** An Indigenous person recognized and identified by the Elders of the community as being knowledgeable about cultural practices, products, or Indigenous world views

**Métis:** the descendants of Indigenous and European settlers who formed mixed communities around the fur trade. The Métis are recognized as Aboriginal peoples in Canada. The term Métis is viewed differently across the country and its use can be complex and contentious.

**Reconciliation:** the restoration of relations between Indigenous and non-Indigenous peoples in Canada through the establishment and maintenance of mutually respectful relationships.

**Reserve:** a portion of land owned by the Canadian government that has been set aside for First Nation peoples.

**Self-determination:** The rights of a community to determine what is best for them. Indigenous peoples have the right of self-determination. By virtue of that right they freely determine their political status and freely pursue their economic, social and cultural development.

**Self-governance:** allows Indigenous nations to build their governance capacity and institutions and take increased control over law-making authority.

**Tradition/traditional:** Cultural practices and institutions that have been followed in the past that are not static but are continually evolving.

**Treaty:** agreements made between two, or possibly even several Nations or governments. Treaties are meant to govern the relationship between parties. Another name for a treaty may be an agreement or covenant.

**Truth and Reconciliation Commission of Canada:** The Truth and Reconciliation Commission was established as part of the Indian Residential Schools Settlement Agreement in 2008. Thousands of survivors, their families and others across Canada made statements to document memories of the schools and their impacts. An Interim Report was released in 2012. The Final Report was delivered in 2015 including 94 recommendations for future action. The statements, documents and other materials are housed at the National Research Centre on Indian Residential Schools at the University of Winnipeg, where the work of the Commission will be carried on.

**Two-Eyed Seeing:** seeing with a traditional (or Indigenous) eye, while acknowledging and seeing with a “western” eye.

Culled from:

1. Younging G. Elements of Indigenous style: A guide for writing by and about Indigenous peoples. Brush Education; 2018 Mar 1. Brush Education Inc.
2. The Nova Scotia Government, Department of Communities, Culture and Heritage and the Department of Labour & Advanced Education. Available from: <https://ions.ca/wp-content/uploads/2020/06/CSCNS-DLJ-Glossary-of-terms.pdf>.
3. The Alberta Teachers’ Association. Walking Together Education for Reconciliation. Available from: <https://www.teachers.ab.ca/SiteCollectionDocuments/ATA/For%20Members/ProfessionalDevelopment/Walking%20Together/PD-WT-16a%20-%20Terminology.pdf>
4. BC First Nations Land, Title, and Governance. Glossary of Terms. Available from: <http://www.fnesc.ca/wp/wp-content/uploads/2019/08/10.-Glossary-1.pdf>
